# A Pseudovirus Nanoparticle-Based Trivalent Rotavirus Vaccine Candidate Elicits High and Cross P Type Immune Response

**DOI:** 10.3390/pharmaceutics14081597

**Published:** 2022-07-30

**Authors:** Ming Xia, Pengwei Huang, Ming Tan

**Affiliations:** 1 Division of Infectious Diseases, Cincinnati Children’s Hospital Medical Center, Cincinnati, OH 45229, USA; ming.xia@cchmc.org (M.X.); pengwei.huang@cchmc.org (P.H.); 2 Department of Pediatrics, University of Cincinnati College of Medicine, Cincinnati, OH 45229, USA

**Keywords:** rotavirus, S_60_-VP8* pseudovirus nanoparticle, rotavirus vaccine, rotavirus VP8*, non-replicating rotavirus vaccine, norovirus S_60_ nanoparticle

## Abstract

Rotavirus infection continues to cause significant morbidity and mortality globally. In this study, we further developed the S_60_-VP8* pseudovirus nanoparticles (PVNPs) displaying the glycan receptor binding VP8* domains of rotavirus spike proteins as a parenteral vaccine candidate. First, we established a scalable method for the large production of tag-free S_60_-VP8* PVNPs representing four rotavirus P types, P[8], P[4], P[6], and P[11]. The approach consists of two major steps: selective precipitation of the S-VP8* proteins from bacterial lysates using ammonium sulfate, followed by anion exchange chromatography to further purify the target proteins to a high purity. The purified soluble proteins self-assembled into S_60_-VP8* PVNPs. Importantly, after intramuscular injections, the trivalent vaccine consisting of three PVNPs covering VP8* antigens of P[8], P[4], and P[6] rotaviruses elicited high and broad immunogenicity in mice toward the three predominant P-type rotaviruses. Specifically, the trivalent vaccine-immunized mouse sera showed (1) high and balanced IgG and IgA antibody titers toward all three VP8* types, (2) high blocking titer against the VP8*-glycan receptor interaction, and (3) high and broad neutralizing titers against replications of all P[8], P[4], and P[6] rotaviruses. Therefore, trivalent S_60_-VP8* PVNPs are a promising non-replicating, parenteral vaccine candidate against the most prevalent rotaviruses worldwide.

## 1. Introduction

Rotaviruses, a group of double-stranded RNA viruses in the family Reoviridae, are causative agents of contagious gastroenteritis in infants and young children with typical symptoms of severe watery diarrhea, vomiting, abdominal pain, and/or fever, often leading to dehydration, and occasional death. Before a vaccine was available, nearly every child was infected with rotavirus at least once by the age of five, with the first infection usually occurring before three years of age [1]. Through the implementation of live attenuated vaccines since 2006, the rotavirus disease burden around the globe has significantly decreased, particularly in developed countries [2,3]. Nevertheless, the effectiveness of oral vaccines has been found to be diminished in source-deprived, low-income nations [4,5]. Consequently, rotavirus-associated diseases continue to cause about 24 million outpatient visits, 2.3 million hospitalizations, and 200,000 deaths worldwide each year [6,7]. Therefore, rotavirus-associated diarrhea remains a global public health threat, and a new generation of rotavirus vaccine tactics with improved efficacy is urgently needed, especially for children in developing countries where rotavirus infection occurs the most.

The reasons underlying the reduced effectiveness of current live rotavirus vaccines in low-income nations are not fully understood [8,9]. The growing literature points to multiple factors that affect the intestinal environment of children in developing countries [9]. These include microbiota dysbiosis [10], malnutrition [11], enterovirus infection [12], and the simultaneous immunization of poliovirus and other oral vaccines [9,13]. These factors may play a role in altering the intestinal conditions necessary for optimal replication of the live rotavirus vaccines, thus negatively impacting the immune responses and the efficacy of the oral vaccines, which are also common to other live, oral vaccines [9].

In summary of related literature, a recent study [14] quantified rotavirus vaccine impact, investigated the reduced vaccine effectiveness using sophisticated mathematical models, and proposed a parenteral vaccine tactic to circumvent the negative impact of the above-mentioned intestine-associated factors. Therefore, a non-replicating subunit vaccine administrated via a parenteral route could enhance rotavirus vaccine efficacy for children in developing nations. In addition, the known risk of intussusception associated with the live rotavirus vaccine [15,16,17,18,19,20,21] may result from the replication of oral vaccines within the intestine. Thus, a non-replicating parenteral vaccine may also avoid intussusception risk, offering an improved safety feature.

Our development of a non-replicating subunit rotavirus vaccine started with the P_24_-VP8* nanoparticle [22,23,24] that consists of a 24 valent P_24_ nanoparticle core made by 24 norovirus protruding (P) domains [25,26] and 24 surface displayed VP8* antigens. The VP8* antigens form the distal heads of rotavirus spikes, which are composed of rotavirus VP4s. Since VP8* interacts with glycan receptors to initiate a viral infection, it is a major neutralizing antigen and thus an important vaccine target of a rotavirus subunit vaccine [23,27,28,29,30,31,32,33,34]. The P_24_-VP8* nanoparticles were shown to elicit high immune responses in mice [23] and pigs [35] towards the displayed VP8* antigens after intramuscular immunization and protected immunized mice and gnotobiotic pigs from rotavirus challenge [23,35]. In this regard, rotavirus-like particles consisting of VP2, VP6, and/or VP7 [36,37], P2-VP8-P[8] fusion proteins composed of a tandem of two VP8* with a T cell epitope in between [38,39], and truncated VP4 trimers [40] have been generated and studied as non-replicating rotavirus vaccine candidates by others.

The rotavirus virion is a triple-layered particle about 85 nm in diameter. It consists of a core shell constituted by VP2, a middle layer formed by VP6, and an outer layer consists of two surface proteins, VP7 and VP4. Rotaviruses are categorized into G and P genotypes based on the gene sequences encoding the surface proteins VP7 and VP4/VP8*, respectively. P[8] and P[4] are the two most prevalent P genotypes [41], contributing to up to 95% of circulated rotaviruses around the world. It has also been noted that P[6] rotaviruses are frequently detected in Africa, accounting for up to 30% of the detected rotaviruses [42,43]. These data indicate that a potent rotavirus vaccine should be able to protect vaccinees against P[8], P[4], and P[6] rotaviruses, particularly for use in developing countries. In addition, although less prevalent than P[8], P[4], and P[6] rotaviruses, the P[11] genotype is often found in India [44,45,46,47]. In fact, India has developed and licensed a live Rotavac^®^ vaccine (Bharat Biotech) that contains a single P[11] rotavirus strain.

In an attempt to create a new non-replicating subunit rotavirus vaccine, we took advantage of our recently developed S_60_ nanoparticle that consists of 60 norovirus shell (S) domains, to generate an S_60_-VP8* pseudovirus nanoparticle (PVNP) that displays 60 copies of VP8* antigen of a P[8] rotavirus on the surface [48]. This polyvalent S_60_-VP8* PVNP preserves the pathogen-associated molecular patterns (PAMPs) of both norovirus and rotavirus and thus induces high immune responses toward the VP8* antigens and protects mice from the rotavirus challenge [33,48,49]. The S_60_-VP8* PVNP was purified using a His tag that appears to reduce the solubility and thus the production yield of the PVNP, which may impose a negative factor in the downstream development of the vaccine candidate. In this study, we designed tag-free S_60_-VP8* PVNPs displaying VP8* antigens representing the four predominant rotavirus P types and developed an efficient, scalable production approach to generate tag-free PVNPs in a large amount. A trivalent vaccine consisting of S_60_-VP8* PVNPs covering VP8* antigens of P[8], P[4], and P[6] rotaviruses elicited high and broad immune responses to all three VP8* types, offering a promising vaccine candidate against predominant rotaviruses circulating globally.

## 2. Materials and Methods

### 2.1. Plasmids for Expression of Four Tag-Free S-VP8* Fusion Proteins

Three DNA fragments that encode the major functional sections of the VP8* domains, spanning from L65 to L223 of rotavirus VP4 proteins of a P[8] (strain 13851), a P[4] (strain BM5256), and a P[6] (strain 11597) virus, respectively, were amplified by PCR from our lab stock plasmids [22,30,50]. The DNA fragments were then cloned into the previously made pET-24b (Novagen)-based vector that was generated for production of the C-terminally His-tagged S_60_-VP8* P[8] PVNP [48] by replacing its VP8* encoding sequences. The S domain-encoding region in the plasmids contains an R69A mutation to remove the exposed protease recognition site [48]. In addition, a DNA fragment encoding the same VP8* region of a P[11] rotavirus (GenBank Code: EU200796) was codon-optimized to *Escherichia coli* (*E. coli*) and synthesized by GenScript (Piscataway, NJ, USA). The synthesized DNA fragment was subcloned into the above-mentioned plasmid using the same approach. A stop codon was added in front of the His tag-encoding sequences of pET 24b to remove the His tag.

### 2.2. Expression and Purification of Tag-Free S-VP8* Fusion Proteins

Tag-free S-VP8* proteins were expressed using the *E. coli* (strain BL21, DE3) system through an induction with 0.25 mM isopropyl-β-D-thiogalactopyranoside (IPTG) at ~22 °C overnight as described elsewhere [25,51]. For protein purification, bacteria were lysed by sonication, and the bacterial lysates were clarified by centrifugation at 10,000 rpm for 30 min using an Avanti J26XP centrifuge (Beckman Coulter Life Sciences, Indianapolis, IN, USA) and a JA-17 rotor. Clarified supernatants were treated with ammonium sulfate [(NH_4_)_2_SO_4_] at 1.2 M end concentrations for 30 min to selectively precipitate the target proteins. The protein precipitations were collected by centrifugation at 5000 rpm for 20 min using the same centrifuge and rotor (see above), washed twice using 1.2 M (NH_4_)_2_SO_4_ solution in 20 mM Tris buffer (pH 8.0), and then dissolved in 20 mM Tris buffer (pH 8.0), as described previously [22].

### 2.3. Anion Exchange Chromatography

Anion exchange chromatography was conducted to further purify the (NH_4_)_2_SO_4_-precipitated S-VP8* proteins using an AKTA Fast Performance Liquid Chromatography System (AKTA Pure 25L, GE Healthcare Life Sciences, Piscataway, NJ, USA) with a HiPrep Q HP 16/10 column (20 mL bed volume, GE Healthcare Life Sciences, Piscataway, NJ, USA), as described previously [22]. Briefly, the column was equilibrated with 5 column volumes (CV) of 20 mM Tris-HCl buffer (pH 8.0, referred to as buffer A). After loading the protein samples (~5 mL), the column was washed using 7 CVs of buffer A. The bound proteins were eluted using 7 CVs 1 M NaCl in buffer A (referred to as buffer B) through a linear gradient (0 to 100% buffer B). The column was washed with 7 CVs of buffer B, followed by a final equilibration with 7 CVs of Buffer A. Relative protein amounts in the effluent were shown by A280 absorbance.

### 2.4. Sodium Dodecyl Sulfate Polyacrylamide Gel Electrophoresis (SDS-PAGE)

SDS-PAGE was performed to analyze the protein quality using 10% separating gels. Protein concentrations were determined by SDS-PAGE using serially diluted bovine serum albumin (BSA, Bio-Rad, Hercules, CA, USA), with known concentrations as standards on the same gels [23].

### 2.5. Transmission Electron Microscopy (TEM)

TEM was performed to inspect the morphology of the S_60_-VP8* PVNPs. PVNP samples from gel filtration chromatography or a CsCl density gradient in 6.0 µL volume were absorbed to a grid (FCF200-CV-50, Electron Microscopy Sciences, Hatfield, PA, USA) for 20 min in a humid chamber and were negatively stained with 1% ammonium molybdate. After washing and air drying, the grids were observed using a Hitachi microscope (model H-7650) at 80 kV for a magnification between 15,000× and 60,000× as described elsewhere [48].

### 2.6. Cesium Chloride (CsCl) Density Gradient Ultracentrifugation

This method was utilized to analyze the density of the S_60_-VP8* PVNPs as described previously [48]. Briefly, 0.5 mL of the purified PVNPs were mixed with CsCl solution to a volume of 10 mL with a density of 1.3630 and centrifuged at 41,000 rpm (288,000× *g*) for 45 h using the Optima L-90K ultracentrifuge (Beckman Coulter Life Sciences, Indianapolis, IN, USA). By bottom puncture of the centrifugation tubes, the CsCl gradients were fractionated into 22 fractions, with about 0.5 mL each. The S-VP8* PVNPs in the fractions were detected by EIA assays after 100-fold dilution in phosphate buffer saline (PBS, pH 7.4) and coated on 96-well microtiter plates (Thermo Scientific, Waltham, MA, USA) using our in-house-made hyperimmune guinea pig serum against norovirus VLPs [52]. The CsCl densities of the fractions were determined using the refractive index.

### 2.7. Mouse Immunization with the S_60_-VP8 PVNPs and Controls

Rotavirus-free BALB/c mice at age about six weeks with body weight ranging from 19 to 23 g were randomly divided into five groups with 6 to 8 mice each (N = 6–8) that were immunized with following immunogens, respectively: (1) the trivalent PVNP vaccine consisting of three S_60_-VP8* PVNPs that display the VP8* antigens of P[8], P[4], and P[6] rotaviruses, respectively, in equal molar ratio at 30 µg/mouse/dose (10 µg of each PVNP type); (2) the S_60_-VP8* PVNPs of P[8] rotavirus at 10 µg/mouse/dose; (3) the S_60_-VP8* PVNPs of P[4] rotavirus at 10 µg/mouse/dose; (4) the S_60_-VP8* PVNPs of P[6] rotavirus at 10 µg/mouse/dose; and (5) the S_60_ nanoparticles without the VP8* antigens [25,26] at 10 µg/mouse/dose as a negative control. All immunogens were treated with endotoxin removal resin (Pierce, Waltham, MA, USA) to remove endotoxin contamination. The immunogens were delivered with an Alum adjuvant (Thermo Scientific, aluminum hydroxide, 40 mg/mL) at 25 μL/dose through 1:1 mixing with immunogens at 20 μg/mouse/dose, as described elsewhere [22]. This resulted in an end aluminum hydroxide dose of 1.0 mg/dose/mouse. Immunogens in 50 μL volumes were injected intramuscularly into the thigh muscle. Immunizations were performed three times at 2-week intervals. Blood samples were taken before the first immunization, as well as two weeks after the second and the third immunization, through tail veins (before the first immunization and after the second immunization) and the heart puncture approach (after the third immunization) for serum sample preparations [23].

### 2.8. Enzyme Immunoassays (EIAs)

EIAs were performed to detect the S-VP8* proteins in the fractions of the CsCl gradients (see above) and to determine the VP8*-specific antibody titers [48]. For antibody determination, gel filtration purified GST-VP8* fusion proteins of P[8], P[4], and P[6] rotaviruses from our lab stock [30,50] were coated on microtiter plates at 1 μg/mL overnight. After blocking with nonfat milk, the plates were incubated with mouse sera at serial 2x dilutions. Bound antibodies were measured with goat-anti-mouse IgG-horse radish peroxidase (HRP) conjugate (1:5000, MP Biomedicals, Santa Ana, CA, USA) for VP8*-specific IgG or goat-anti-mouse IgA-horse radish peroxidase (HRP) conjugate (1:2000, Invitrogen, Waltham, MA, USA) for VP8*-specific IgA. Antibody titers were defined as the maximum dilutions of sera that exhibited at least cut-off signals of OD_450_ = 0.15, as described previously [48].

### 2.9. 50% Blocking Titer (BT_50_) of Sera against Rotavirus VP8*-Glycan Receptor Attachment

This was determined as described previously [53]. Briefly, well-characterized human saliva samples with Lewis b (Le^b^) antigens from our lab stock [52] were boiled and coated on microtiter plates at 1:1000 dilution. The P_24_-VP8* nanoparticle at 0.625 μg/mL was pre-incubated with the PVNP-immunized mouse sera at different dilutions before the P_24_-VP8* nanoparticles were added to the coated saliva samples. The BT_50_ was defined as the serum dilutions that caused at least 50% blocking effects compared with the unblocked positive controls.

### 2.10. Rotavirus Neutralization Assays

This fluorescence-based plaque reduction assay was performed as described previously [22,49]. Briefly, rotaviruses of the P[8] (Wa strain, G1P8), P[6] (ST-3 strain, G4P6), and P[4] (DS-1 strain, G2P4) types were treated with trypsin and incubated with serially diluted mouse sera after various PVNP immunizations. The treated rotaviruses were then added to the MA104 cells on 96-well plates, and the cells were continually cultured for 16 h. The cells on the plates were then fixed with pre-cooled 80% (*v*/*v*) acetone, followed by blocking with nonfat milk. The rotavirus-infected cells were stained with guinea pig antiserum (1:800) against rotaviruses. The bound antibodies were shown by fluorescein DyLight 594-labeled goat anti-guinea pig IgG (H +L) antibodies (Jackson Immuno Research Labs, West Grove, PA, USA). Fluorescence-formation plaques on plates were photographed using a Cytation 5 imaging reader, and fluorescence plaques (rotavirus-infected cells) were counted. Neutralization titers were described as the maximum dilutions of the mouse sera, showing at least a 50% reduction in fluorescence-formation plaques compared with the positive control with mouse serum.

### 2.11. Statistical Analyses

Statistical differences between data groups were analyzed using software GraphPad Prism version 9.3.1 (471) (GraphPad Software, Inc., San Diego, CA, USA) via unpaired *t* tests. Differences were classified as follows: (1) non-significant (labeled as ns), when a *p* value is >0.05, (2) significant (labeled as *), when a *p* value is <0.05, (3) highly significant (labeled as **), when a *p* value is <0.01, and (4) extremely significant, when a *p* value is <0.001 (labeled as ***), or <0.0001 (labeled as ****).

## 3. Results

### 3.1. Expression and Selective Precipitation of the S-VP8* Proteins

**Figure 1:**
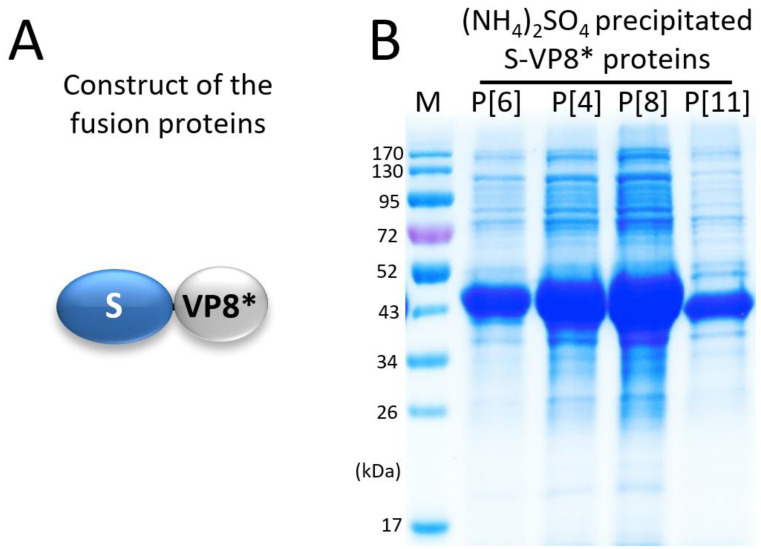
Expression and selective precipitation of the S-VP8* fusion proteins. (**A**) Schematic diagram of the S-VP8* fusion proteins. S, modified norovirus shell (S) domain; VP8*, rotavirus VP8* domain. (**B**) SDS-PAGE of the ammonium sulfate [(NH_4_)_2_SO_4_] precipitated S-VP8* fusion proteins at about 43 kDa, which contain the VP8* domains of P[6], P[4], P[8], and P[11], respectively. Lane M is a pre-stained protein standards with indicated molecular weights in kDa.

Four S-VP8* fusion proteins (Figure 1A), each containing the VP8* domain of a P[4], a P[6], a P[8], or a P[11] rotavirus, were expressed using the *E. coli* system. After IPTG induction, bacterial cultures were collected and sonicated to release soluble S-VP8* proteins. The clarified bacterial lysates were treated with 1.2 M (NH_4_)_2_SO_4_ to selectively precipitate the target proteins, which were then dissolved in 20 mM Tris buffer (pH 8.0). The S-VP8* proteins at about 43 kDa were shown on an SDS-PAGE gel (Figure 1B), revealing the S-VP8* proteins as the major precipitated proteins, with a number of co-precipitated bacterial proteins.

### 3.2. Purification of S-VP8* Proteins by Ion Exchange Chromatography

**Figure 2:**
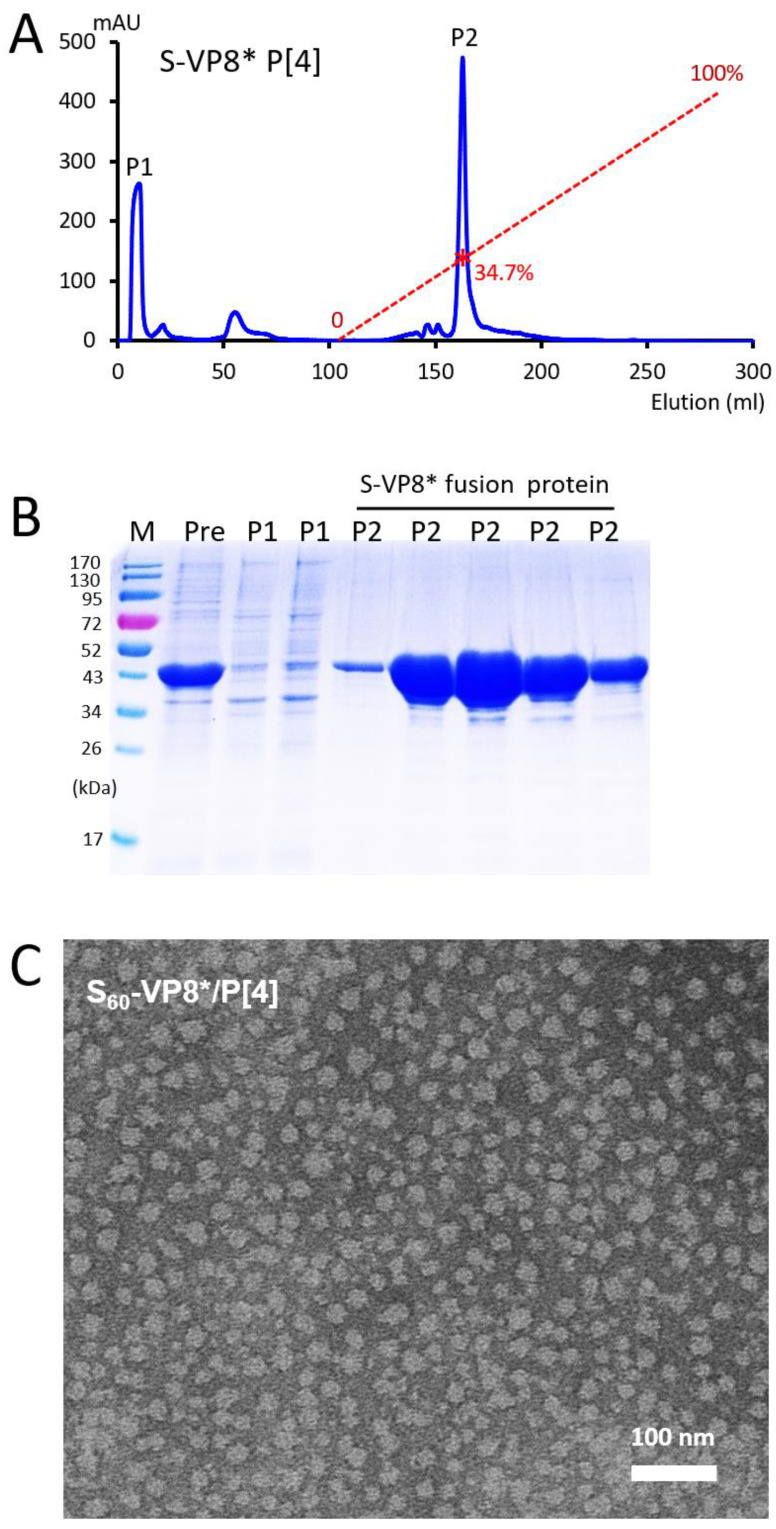
Purification of tag-free S-VP8* P[4] protein and its self-assembly into pseudovirus nanoparticles (PVNPs). (**A**) An anion exchange elution curve of the ammonium sulfate [(NH_4_)_2_SO_4_] precipitated S-VP8* P[4] protein. The *X*-axis indicates elution volume (mL), whereas the *Y*-axis shows UV (A_280_) absorbances (mAU). The red dashed line indicates the linear increase of elution buffer B (0–100%), with a red star symbol indicating the percentage of buffer B at the elution peak of the S-VP8* P[4] protein (34.7%). Two major peaks that were analyzed by SDS-PAGE are indicated as P1 and P2. (**B**) SDS-PAGE of the pre-loaded protein (Pre), as well as proteins from peak 1 (P1) and peak 2 (P2) from the anion exchange chromatography. Pre is the (NH_4_)_2_SO_4_ precipitated protein samples before loading to the column; M is the pre-stained protein standards with indicated molecular weights in kDa. The S-VP8* P[4] protein was eluted in P2. (**C**) A micrograph of negative-staining transmission electron microscopy (TEM) of the protein from P2 shows spheric-shaped PVNPs.

The (NH_4_)_2_SO_4_ precipitated S-VP8* proteins of the four rotavirus P types were further purified using anion exchange chromatography, with their elusion curves shown in Figure 2A (P[4]), Figure S1A (P[6]), Figure S2A (P[8]), and Figure S3A (P[11]), respectively. The major elution peaks of the corresponding chromatography were analyzed by SDS-PAGE (Figure 2B, Figure S1B, Figure S2B and Figure S3B), showing three common features among these four ion exchanges. First, the vast majority of the co-precipitated bacterial proteins did not bind or did not bind well to the HiPrep Q HP 16/10 column. As a result, they either flowed directly through the column or were washed away during the washing step afterwards. Second, all four target proteins were eluted in single narrow peaks with relatively low salt concentrations, corresponding to 34.7% to 38.7% of buffer B, equivalent to 347 mM to 387 mM NaCl. Third, the S-VP8* proteins were separated well from the co-precipitated bacterial proteins, reaching to high yields of the proteins at >30 mg/liter of bacterial culture and a high purity of >95% (Figure 2B, Figure S1B, Figure S2B and Figure S3B). Thus, this production method serves as a simple but effective and scalable approach for low-cost production of tag-free PVNPs (see below).

### 3.3. Self-Formation of Purified S-VP8* Proteins into PVNPs

The ion exchange chromatography purified S-VP8* fusion proteins were inspected by negative strain TEM, revealing large numbers of nanoparticles at about 25 nm in diameter, with some size variations (Figure 2C, Figure S1C, Figure S2C and Figure S3C). Since the previously solved 3-dimensional structure of the His tagged S_60_-VP8 P[8] PVNPs exhibited about 25 nm in diameters [48], these micrographs indicated that the four tag-free S-VP8* fusion proteins self-assembled into S_60_-VP8* PVNPs. The size variations disappeared after the PVNPs were further analyzed by CsCl gradient ultracentrifugation (see below).

### 3.4. CsCl Gradient Ultracentrifugation of the PVNPs

**Figure 3:**
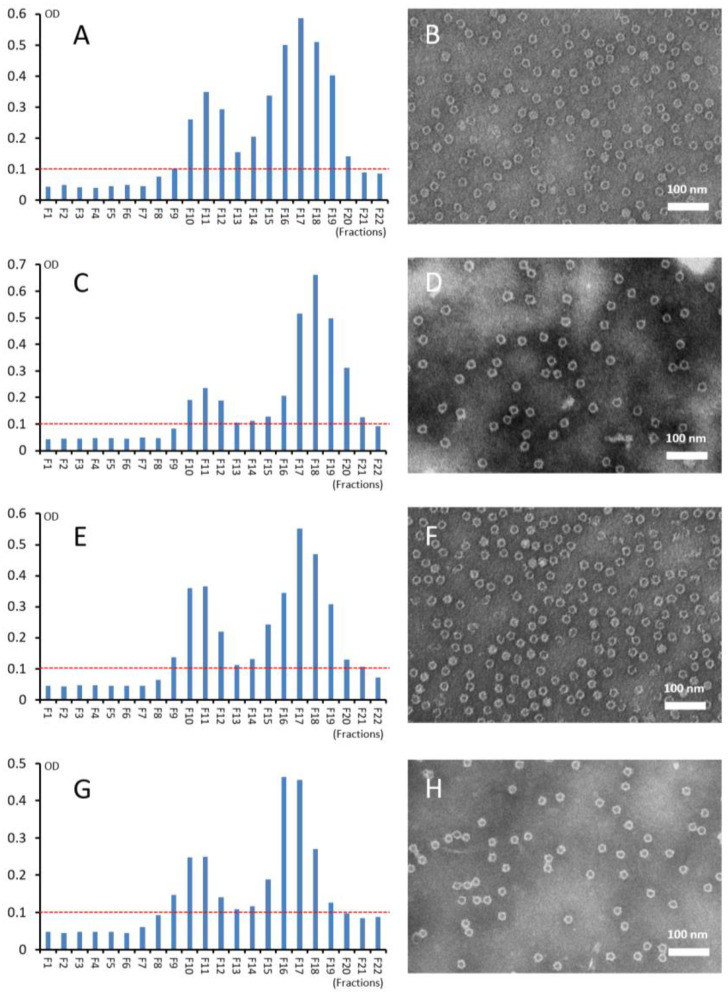
Analyses of the four S_60_-VP8* PVNPs using cesium chloride (CsCl) density gradient centrifugation and transmission electron microscopy (TEM). (**A**,**C**,**E**,**G**) Following centrifugation, the CsCl density gradients containing the S_60_-VP8* PVNPs of P[4] (**A**), P[6] (**C**), P[8] (**E**), and P[11] (**G**) rotaviruses, respectively, were fractionated into 22 portions. The relative S_60_-VP8* protein amounts in the fractions were measured by EIA assays using a hyperimmune antibody against norovirus VLP [52]. Y-axes show signal intensities in optical density (OD), with red dashed lines showing the cut-off signal at OD = 0.1, while X-axes indicate the fraction numbers. (**B**,**D**,**F**,**H**) Negative stain TEM micrographs of the S_60_-VP8* PVNPs from fractions 17 (**B**,**F**,**H**), or 18 (D), showing uniform, ring-shaped PVNPs containing VP8* antigens of the P[4] (**B**), P[6] (**D**), P[8] (**F**) rotaviruses, and P[11] (**H**) rotaviruses, respectively.

The four S-VP8* fusion proteins were analyzed by CsCl gradient ultracentrifugation, which revealed a major and minor peak for each S-VP8* fusion protein (Figure 3A,C,E,G left panel). The major peaks were located at or near fraction 17, whereas the minor peaks were at fraction 11 of the gradients, equivalent to densities of 1.305 and 1.311 mg/cm^3^, respectively. TEM inspection of the protein samples from the major peaks (fractions 17 or 18) showed uniform, ring shaped S_60_-VP8* PVNPS (Figure 3B,D,F,H right panel). Inspection of the proteins from the minor peaks (fraction 11) also showed similar ring-shaped PVNPs, but the PVNP numbers were apparently less abundant (data not shown) than those of the major peaks. It remains unclear why similar PVNPs are distributed in two distinct peaks with different densities.

### 3.5. Trivalent PVNP Vaccine and Its IgG Responses in Mice

Surveillance data showed that P[8], P[4], and P[6] rotaviruses are the predominant rotavirus P types [41,42,43], thus three S_60_-VP8* PVNP types, displaying the VP8* antigens of P[8], P[4], and P[6] rotaviruses, respectively, were mixed at equal molar ratios as a trivalent vaccine. Mice were immunized intramuscularly with the trivalent vaccine, using aluminum hydroxide as an adjuvant. The three individual PVNPs were used as single-valent PVNP vaccine controls, and the S_60_ nanoparticle without the VP8* antigen was used as a platform control for comparisons (see Materials and methods). Serum samples were collected after two and three immunizations to determine the VP8*-specific antibody responses by EIA assays using recombinant VP8* proteins of P[8], P[4], and P[6] rotaviruses, respectively, as capture antigens.

**Figure 4:**
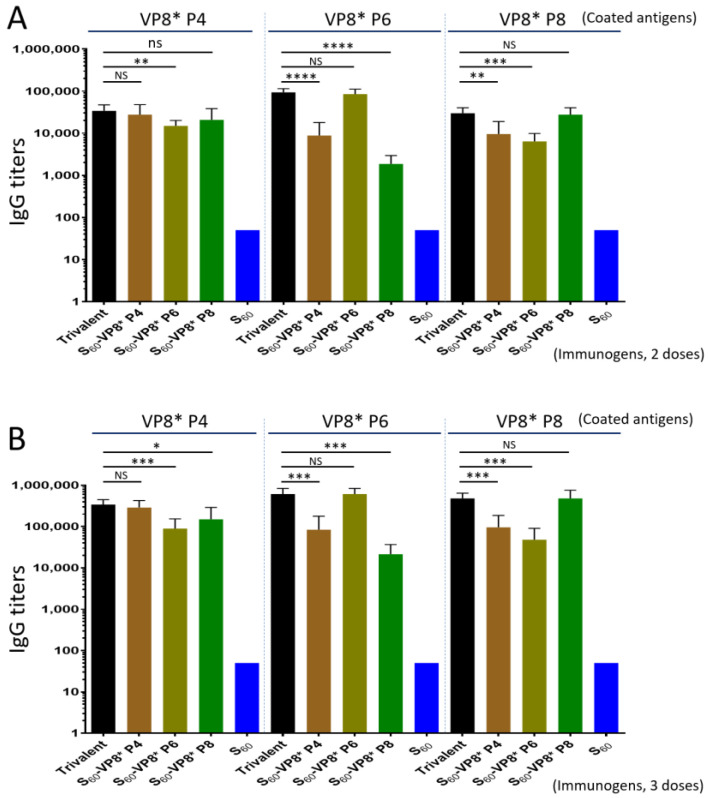
The trivalent S_60_-VP8* PVNP vaccine (trivalent) induced higher and broader IgG responses in mice toward the three homologous VP8* antigens (black columns) compared with those elicited by the three single-valent S_60_-VP8* PVNPs (brawn/cyan/green columns) after two (**A**) and three (**B**) immunizations. The *Y*-axis shows the VP8*-specific IgG titers, while the *X*-axis shows different vaccines or immunogens, as indicated. Statistical differences between data groups with corresponding *p*-values are calculated and shown in the Supplementary Materials. “NS”, non-significant for *p*-values > 0.05; “*”, significant for *p*-values < 0.05; “**”, highly significant for *p*-values < 0.01; “***”, extremely significant for *p*-values < 0.001; “****”, extremely significant for *p*-values < 0.0001.

The results (Figure 4) showed that, after three immunizations, the trivalent vaccine induced high and balanced IgG titers (341,333 to 614,533) to all P[8], P[4], and P[6] VP8*s to levels similar to those elicited by the single-valent PVNPs against their homologous VP8* antigens (*Ps* > 0.05, Figure 4B). It was noted that the sera after immunization with the individual single-valent PVNPs showed significantly lower IgG titers against the two heterologous VP8* antigens (*Ps* < 0.05, Figure 4B). Similar scenarios of immune responses of the trivalent vaccine and individual PVNP and S_60_ controls were also observed after two immunizations (Figure 4A), but the resulting IgG titers were significantly lower compared with those after three immunizations (*Ps* < 0.05), which was expected. We also noted that the sera after immunizations with the single-valent P[8] or P[4] PVNP revealed higher cross-reactivity against each other of the two VP8* antigens compared with that against the P[6] VP8* antigen. These observations reflect their evolutionary distances, as P[8] and P[4] rotaviruses are members of the same genetic lineage in the P[II] genogroup; in contrast, P[6] rotavirus belongs to another lineage of the same P[II] genogroup [54]. The S_60_ nanoparticle without VP8* an antigen did not elicit detectable VP8*-specific IgG (<25).

### 3.6. Serum IgA Responses of the Trivalent PVNP Vaccine

**Figure 5:**
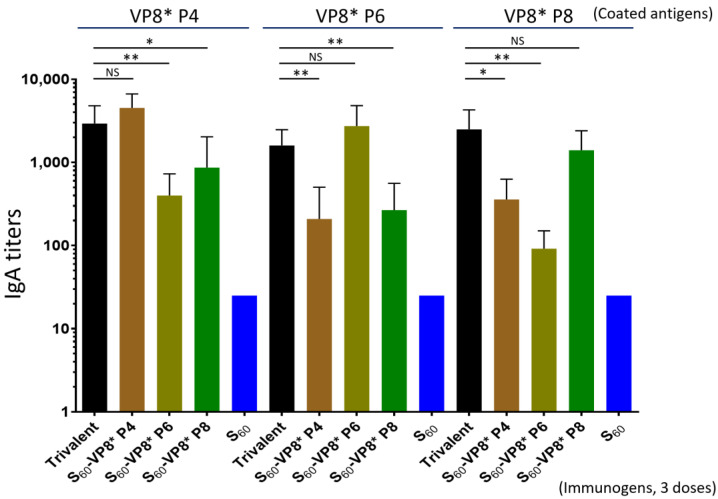
The trivalent S_60_-VP8* PVNP vaccine (trivalent) induced higher and broader serum IgA responses in mice toward the three homologous VP8* antigens (black columns) compared with those elicited by the three single-valent S_60_-VP8* PVNPs (brawn/cyan/green columns) after three immunizations. The *Y*-axis shows the VP8*-specific serum IgA titers, while the *X*-axis shows different vaccines or immunogens, as indicated. Statistical differences between data groups with corresponding *p*-values are calculated and shown in the Supplementary Materials. “NS”, non-significant for *p*-values > 0.05; “*”, significant for *p*-values < 0.05; “**”, highly significant for *p*-values < 0.01.

The mouse serum IgA titers after three immunizations of the trivalent PVNP vaccine, as well as the three single-valent PVNP vaccines, were also determined by EIAs (Figure 5). The outcomes showed that the trivalent vaccine elicited broad IgA titers (1600 to 2933) toward all P[8], P[4], and P[6] VP8* antigens, resembling those induced by the individual single-valent PVNP vaccines against their homologous VP8* antigens (*Ps* > 0.05, Figure 5). Like the IgG responses (Figure 4), the sera after immunization with each single-valent PVNP vaccine showed substantially lower IgA titers against the two heterologous VP8* antigens (*Ps* < 0.05, Figure 5). As expected, the S_60_ nanoparticle without VP8* antigens did not induce detectable VP8*-specific IgA (<12.5). The serum IgA titers after two immunizations of the trivalent PVNP vaccine were not determined due to a lack of sufficient serum samples after they were used for IgG titer determinations.

### 3.7. BT_50_ of the Trivalent Vaccine-Immunized Mouse Sera against VP8*-Glycan Receptor Attachment

**Figure 6:**
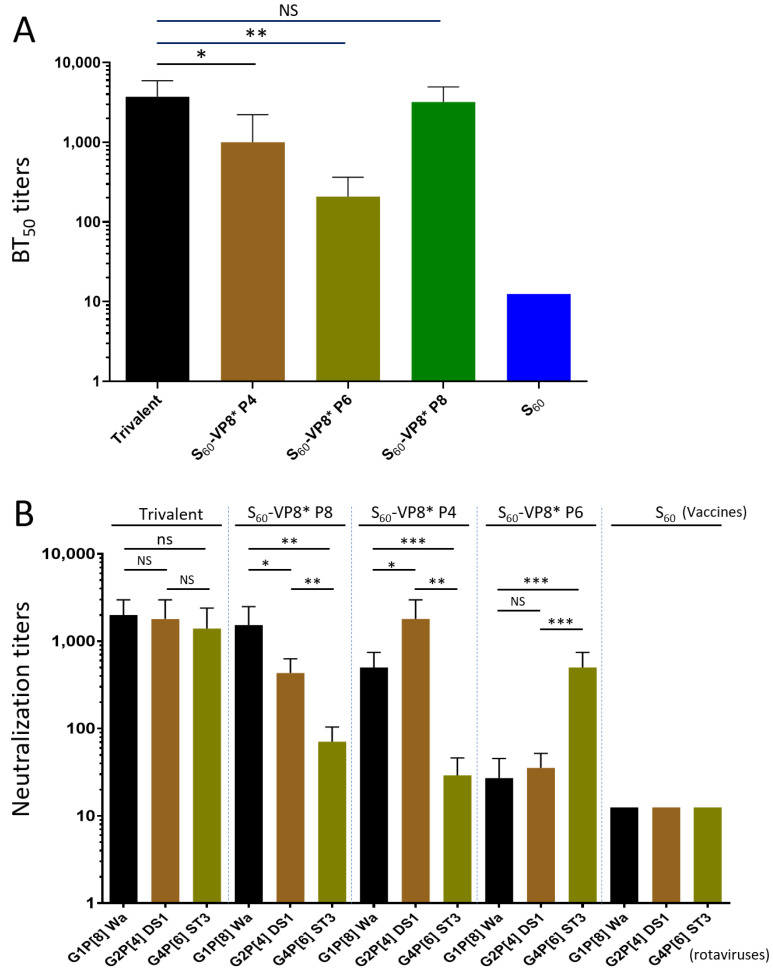
Blocking (**A**) and neutralization (**B**) titers of the mouse sera after immunization with the trivalent PVNP vaccine and controls against predominant rotavirus P types. (**A**) 50% blocking titers (BT_50_, *Y*-axis) of the mouse sera after immunization with the trivalent PVNP vaccine (trivalent) and the three single-valent PVNPs (*X*-axis) against attachment of the P_24_ nanoparticle displaying P[8] VP8* to glycan receptors in a Le^b^ positive saliva sample. (B) 50% neutralization titers (*Y*-axis) of mouse sera after administration with the trivalent vaccine (trivalent) against replication of a P[8] (Wa strain, G1P8, black columns), a P[4] (DS-1 strain, G2P4, brown columns), and a P[6] (ST-3, G4P6, green columns) rotavirus, respectively, in cell culture, using the mouse sera after immunization with the three single-valent PVNPs and the S_60_ nanoparticle without VP8* antigens (S_60_) as controls (indicated on the top). Statistical differences between data groups with corresponding *p*-values are calculated and shown in the Supplementary Materials. “NS”, non-significant for *p*-values > 0.05; “*”, significant for *p*-values < 0.05; “**”, highly significant for *p*-values < 0.01; “***”, extremely significant for *p*-values < 0.001.

The VP8* domain of P[8] rotavirus binds Le^b^ glycans for viral infection [30,50,55] and a blocking assay using the P_24_-VP8* nanoparticle as rotavirus VP8* surrogates and Le^b^ positive saliva samples as Le^b^ glycan source have been developed and used as a surrogate neutralization assay [48,53]. Here, we determined the BT_50_ of the trivalent vaccine-immunized mouse sera against P_24_-VP8* P[8]-glycan receptor attachment using the three individual single-valent PVNP-immunized sera as controls for comparisons. The results (Figure 6A) showed that the trivalent vaccine-immunized mouse sera exhibited a high BT_50_ titer (3733) against the P_24_-VP8* P[8]-Le^b^ interaction, which was similar to that (3200) of the sera after immunization with the single-valent S_60_-VP8* P[8] PVNP (*P* > 0.05). Corresponding to their IgG and IgA titers, the sera after immunization with the single-valent S_60_-VP8* P[4] or S_60_-VP8* P[6] PVNP revealed significantly lower BT_50_ (1000 or 208, *Ps* < 0.05) compared with that of the sera after immunization with the trivalent PVNP vaccine. The sera after immunization with the S_60_ nanoparticle without VP8* antigens did not show detectable blocking activity (<12.5).

### 3.8. Neutralization of the Trivalent Vaccine-Immunized Mouse Sera

The mouse sera after immunization with the trivalent PVNP vaccine were determined for their 50% neutralization titers through fluorescence plaque reduction assays. This revealed high neutralization titers against all three homologous rotavirus strains representing the predominant P[8] (Wa strain, G1P8), P[4] (DS-1, G2P4), and P[6] (ST-3, G4P6) types, reaching titers of 2000, 1800, and 1400, respectively (Figure 6B, *Ps* > 0.05 among the three titers). It was noted that the two titers against P[8] and P[4] rotaviruses were similar to that of sera after immunization with the individual P[8] or P[4] PVNPs against their homologous rotaviruses (*Ps* > 0.05), but the titer against P[6] rotavirus appeared significantly higher than that of sera after immunization with the single-valent P[6] PVNP against the homologous P[6] rotavirus (*Ps* < 0.05), suggesting a potential antigen sparing effect of the trivalent vaccine against P[6] rotavirus.

Unlike the trivalent vaccine that elicited high and balanced neutralization titers against all three P type rotaviruses, which were consistent with their high and broad IgG and IgA responses, the sera after immunization with the individual single-valent PVNPs showed significantly lower neutralization titers against the two heterologous rotaviruses (Figure 6B, *Ps* < 0.05). In addition, corresponding to their evolutionary distances among P[8], P[4], and P[6] rotaviruses (see above), the sera after immunizations with the single-valent P[8] or P[4] PVNP vaccines exhibited higher cross-neutralization titers against each other of the two rotaviruses compared with that against the P[6] rotavirus. These data provide strong evidence that trivalent S_60_-VP8* PVNPs are a promising candidate vaccine against major rotavirus P types circulating around the world.

## 4. Discussion

This report represents a new advancement of our long-term efforts to develop a non-replicating subunit rotavirus vaccine for parenteral administration to circumvent the intestinally related issues associated with the current live, oral rotavirus vaccines. We were able to generate His-tagged S_60_-VP8* PVNPs displaying the VP8* antigens of a P[8] rotavirus previously [33,48]. In this study, based on the success of generating tag-free P_24_-VP8* nanoparticles [22], we developed a scalable approach for large productions of four tag-free S_60_-VP8* PVNPs covering the VP8* antigens of the three predominant rotavirus P types, P[8], P[4], and P[6], as well as a minor P[11] rotavirus, respectively. This was a major improvement because it provides a low-cost production method for our vaccine candidates. Importantly, after intramuscular immunizations, the trivalent vaccine consisting of the three PVNPs displaying the P[8], P[4], and P[6] VP8* antigens induced a high and balanced immune response in mice against all three VP8* antigens. The resulting mouse sera exhibited high and broad neutralization titers against replications of all P[8], P[4], and P[6] rotaviruses. By contrast, the single-valent PVNPs elicited higher titers of IgG, IgA, and neutralization antibodies against the homologous VP8* but significantly lower titers against the two heterologous VP8* antigens and rotaviruses. Therefore, trivalent PVNPs represent a promising non-replicating rotavirus vaccine candidate for parenteral immunization.

The method developed in this study for S_60_-VP8* PVNP production is relatively simple and scalable for the future manufacturing of the vaccine product on an industrial scale. The procedure consists of two major steps: a selective chemical precipitation of the S-VP8* fusion protein from the bacterial lysates, followed by an ion exchange chromatography to further purify the target proteins. Compared with the method that we developed previously to produce the tag-free P_24_-VP8* nanoparticle [22], the one established in this study was apparently more efficient, reaching higher PVNP yields at higher purity. The main reason for this improvement was that the vast majority of the co-precipitated bacterial proteins by ammonium sulfate did not bind or bound only weakly to the ion exchange column. Consequently, the vast majority of the co-precipitated bacterial proteins flow through the column directly or were washed out by the washing step afterwards. This feature led to a scenario in which nearly no contaminated proteins were found in the elution peaks of the target proteins. This method worked well in producing all four tested PVNPs, providing a low-cost approach for the large production of our trivalent rotavirus vaccine candidate. In addition, our data strongly suggest that the method can be applied to generate S_60_-VP8* PVNPs of other P-type rotaviruses.

Due to the propensity of the norovirus shell (S) protein to self-assemble into the S_60_ nanoparticles, the His-tagged S-VP8* fusion protein spontaneously forms the S_60_-VP8* PVNPs spontaneously [48]. The tag-free S-VP8* protein apparently retained this feature and thus assembled into PVNPs automatically, as shown by the negative stain TEM inspections. It was noted that the PVNPs from different steps of the purification procedure exhibited variable morphologies under TEM. Those from an anion exchange column appeared to be in spheric shapes with variations in sizes. However, they showed uniform ring shapes after CsCl density gradient ultracentrifugation. It is not clear whether the specific buffer conditions with presence of CsCl play a role in these morphological differences under TEM. Future studies may be necessary to clarify this phenomenon. In addition, after CsCl density gradient ultracentrifugation, the S-VP8* proteins formed two peaks differing in their densities, but similar PVNPs were seen in both peaks. Thus, it remains elusive for reasons behind the formation of the two peaks in the CsCl density gradient ultracentrifugation.

Literature has shown that anti-rotavirus serum IgA [56,57] and IgG [58] titers, as well as serum neutralizing antibody titers [57] are correlated with rotavirus vaccine efficacy or protection against rotavirus infection. Another study [59] showed that anti-rotavirus serum IgA titers were correlated with IgA titers in the intestines. In a previous study [22] to investigate the immunogenicity of the trivalent P_24_-VP8* nanoparticle vaccine candidate, the same dosage of antigens with the same adjuvant, as well as the same intramuscular immunization routes as those in this study, were used. These similar conditions allowed us to compare the immune responses elicited by the P_24_-VP8* nanoparticles and the S_60_-VP8* PVNPs. We noted that the IgG titers elicited by the trivalent S_60_-VP8* PVNP vaccine (341,333 to 614,533) in this study were at least 3.5-fold higher than those (95,600 to 128,000) induced by the trivalent P_24_-VP8* nanoparticle vaccine in the earlier study [22], after a three-dose immunization (*Ps* < 0.05). Accordingly, the neutralization titers of the sera after immunization with the trivalent S_60_-VP8* PVNP vaccine (1400 to 2000) in this study were over 4.0-fold higher than those (346 to 362) induced by the trivalent P_24_-VP8* nanoparticle vaccine in the previous study [22] (*Ps* < 0.05). Similar higher IgG titers elicited by the individual single-valent S_60_-VP8* PVNPs than those induced by the individual single-valent P_24_-VP8* nanoparticles were also observed (*Ps* < 0.05). The significantly higher immune responses induced by the trivalent S_60_-VP8* PVNP vaccine than those elicited by the trivalent P_24_-VP8* nanoparticle vaccine were most likely due to the greater valences of the S_60_-VP8* PVNPs than those of the P_24_-VP8* nanoparticle (60 vs. 24 valences). In addition, the S_60_ nanoparticle resembling the inner shell of the norovirus capsid may retain better pathogen-specific molecular patterns (PSMPs) than those of the artificially made P_24_ nanoparticles [25,26]. Finally, the adjuvant effects of the S_60_ shell may be stronger than those of the P_24_ nanoparticle. All three factors may contribute more or less to the higher immunogenicity of the trivalent S_60_-VP8* PVNP vaccine than that of the trivalent P_24_-VP8* nanoparticle vaccine.

Unlike their high effectiveness in developed countries, current live rotavirus vaccines show reduced efficacy in low-income nations [4,5], where rotavirus infection occurs the most. As a result, rotavirus infection continues to cause significant morbidity and mortality in developing countries [6,7], even with the implementation of the live virus. The literature shows that intestine-related factors contribute to reduced vaccine efficacy [9,10,11,12,13], because these factors may change intestinal conditions needed for optimal replication of live rotavirus vaccines, leading to reduced vaccine immune responses and efficacies [9,14]. In addition, the risk of intussusception associated with live vaccines [15,16,17,18,19,20,21] may also result from vaccine rotavirus replication in the intestine. Thus, a non-replicating, parenteral vaccine may help circumvent the intestine-related issues of the live vaccines for improved vaccine efficacy in developing nations [14] and our nanoparticle-based subunit vaccine may serve as an excellent choice in this direction. In particular, our trivalent S_60_-VP8* PVNP vaccine containing VP8* antigens of P[8], P[4], and P[6] rotaviruses elicited high and broad immune responses to the predominant rotavirus P types, offering broad immunity against the most prevalent rotaviruses worldwide.

## 5. Conclusions

Our results in this investigation showed that the trivalent S_60_-VP8* PVNP vaccine covering P[8], P[4], and P[6] rotavirus antigens is a promising non-replicating subunit rotavirus vaccine candidate for parenteral administration for broad immunity against predominant rotaviruses around the world.

## Data Availability

Not applicable.

## Supplementary Materials

The following supporting information can be downloaded at: https://www.mdpi.com/article/10.3390/pharmaceutics14081597/s1, Figure S1: Purification of tag-free S-VP8* P[6] protein and its self- assembly into pseudovirus nanoparticles (PVNPs); Figure S2: Purification of tag-free S-VP8* P[8] protein and its self-assembly into pseudovirus nanoparticles (PVNPs); Figure S3: Purification of tag-free S-VP8* P[11] protein and its self-assembly into pseudovirus nanoparticles (PVNPs); Statistical analyses for Figure 4; Statistical analyses for Figure 5; and Statistical analyses for Figure 6.
