# Supplementary material for: A Pseudovirus Nanoparticle-Based Trivalent Rotavirus Vaccine Candidate Elicits High and Cross P Type Immune Response
Source: Pharmaceutics. 2022 Jul 30;14(8):1597. doi: 10.3390/pharmaceutics14081597 (PMC9413348; doi:10.3390/pharmaceutics14081597)
Supplement: Supplementary file 1 [file pharmaceutics-14-01597-s001.zip › pharmaceutics-1825995-supplementary.pdf]

Supplementary Materials for

A Pseudovirus Nanoparticle-Based Trivalent Rotavirus Vaccine Candidate Elicits High and Cross P Type Immune Response

Ming Xia [1§], Pengwei Huang [1§], and Ming Tan [1,2*]

Correspondence to: Ming.Tan@CCHMC.org

This PDF file includes:

Figures. S1 to S3
Statistical analyses for Figures 7 to 9

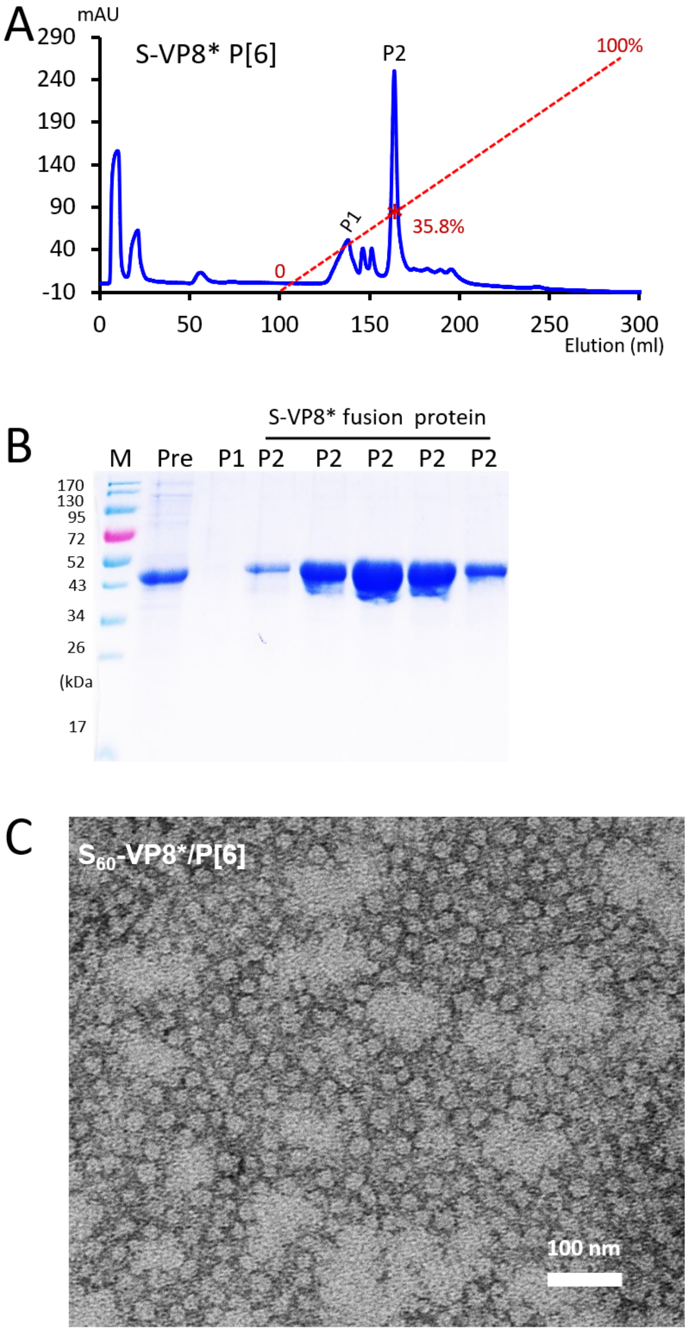

Figure S1. Purification of tag-free S-VP8* P[6] protein and its self- assembly into pseudovirus nanoparticles (PVNPs). (A) An anion exchange elution curve of the ammonium sulfate [$(NH_4)_2SO_4$] precipitated S-VP8* P[6] protein. X-axis indicates elution volume (mL), whereas Y-axis shows UV (A_{280}) absorbances (mAU). The red dashed line indicates the linear increase of elution buffer B (0-100%) with a red star symbol indicating the percentage of buffer B at the elution peak of the S-VP8* P[6] protein (35.8%). Two major elution peaks after applying buffer B that were analyzed by SDS-PAGE are indicated as P1 (peak 1) and P2 (peak 2). (B) SDS-PAGE analysis of the pre-loaded protein (Pre) and samples of P1 and P2 from the ion exchange chromatography. Pre is the $(NH_4)_2SO_4$ precipitated protein sample before loading to the column. M is prestained protein standards with indicated molecular weights in kDa. The S-VP8* P[6] protein was eluted in P2. (C) A micrograph of negative staining transmission electron microscopy (TEM) of the protein from P2 shows spheric-shaped PVNPs.

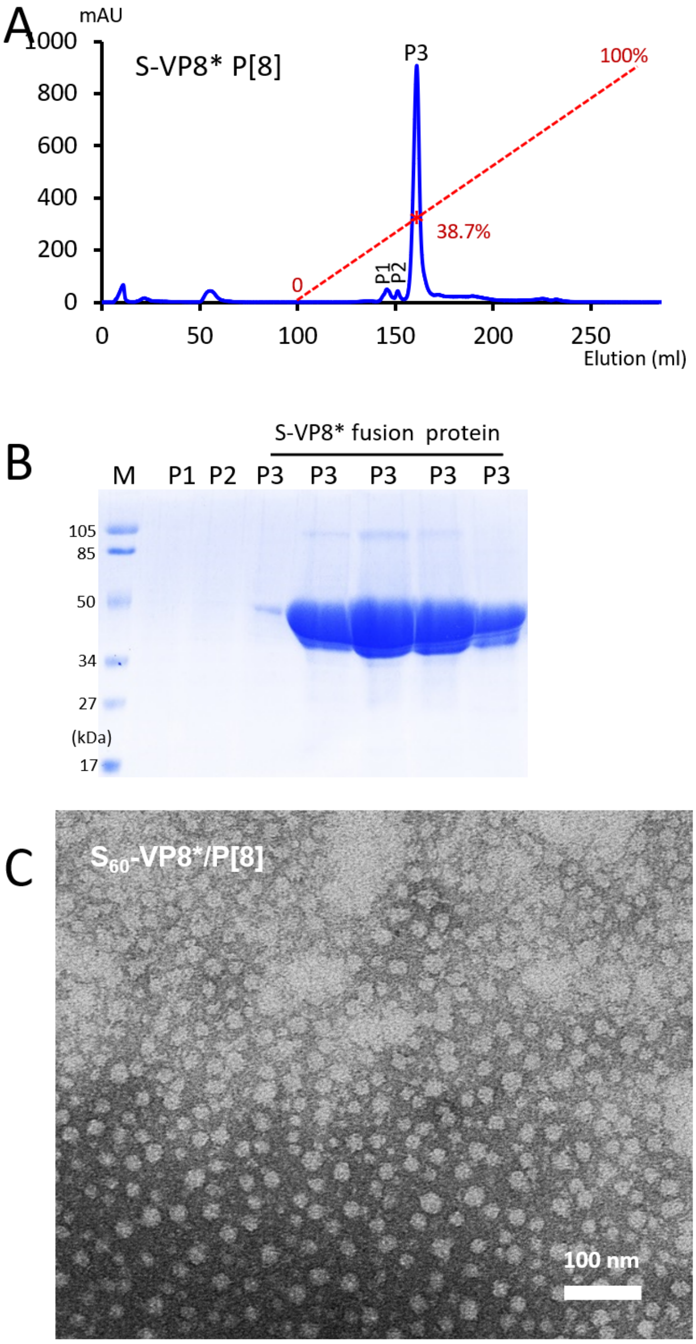

Figure S2. Purification of tag-free S-VP8* P[8] protein and its self-assembly into pseudovirus nanoparticles (PVNPs). (A) An anion exchange elution curve of the ammonium sulfate [$(NH_4)_2SO_4$] precipitated S-VP8* P[8] protein. X-axis indicates elution volume (mL), whereas Y-axis shows UV (A_{280}) absorbances (mAU). The red dashed line indicates the linear increase of the elution buffer B (0-100%) with a red star symbol indicating the percentage of buffer B at the elution peak of the S-VP8* P[8] protein (38.7%). Three elution peaks (P1, P2, and P3) after applying buffer B that were analyzed by SDS-PAGE are indicated. (B) SDS-PAGE analysis of the P1, P2, and P3 elution peaks from the ion exchange chromatography. M is prestained protein standards with indicated molecular weights in kDa. The S-VP8* P[8] protein was eluted in P3. (C) A micrograph of negative staining transmission electron microscopy (TEM) of the protein from P3 peak shows spheric-shaped PVNPs.

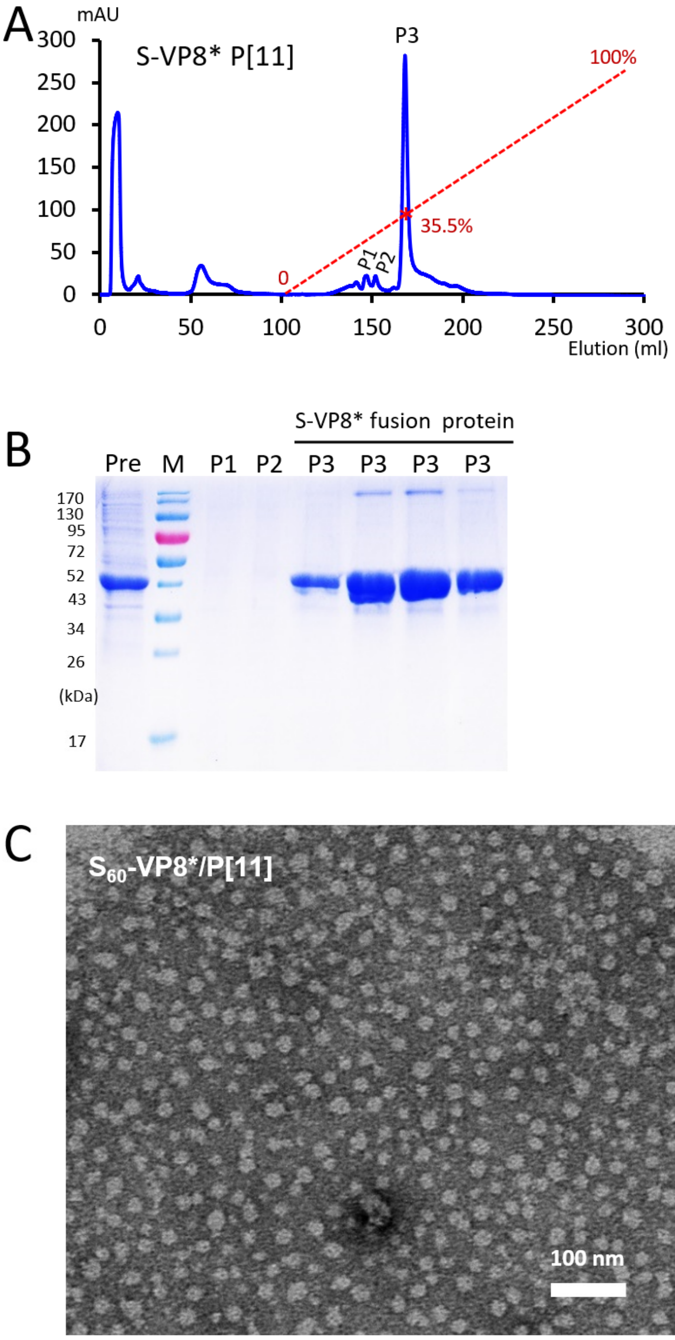

Figure S3. Purification of tag-free S-VP8* P[11] protein and its self-assembly into pseudovirus nanoparticles (PVNPs). (A) An anion exchange elution curve of the ammonium sulfate [$(NH_4)_2SO_4$] precipitated S-VP8* P[11] protein. X-axis indicates elution volume (mL), whereas Y-axis shows UV (A_{280}) absorbances (mAU). The red dashed line indicates the linear increase of the elution buffer B (0-100%) with a red star symbol indicating the percentage of buffer B at the elution peak of the S-VP8* P[8] protein (35.5%). Three elution peaks (P1, P2, and P3) after applying buffer B that were analyzed by SDS-PAGE are indicated. (B) SDS-PAGE analysis of the pre-loaded protein (Pre) and the P1, P2, and P3 elution peaks from the ion exchange chromatography. Pre is the $(NH_4)_2SO_4$ precipitated protein sample before loading to the column. M is prestained protein standards with indicated molecular weights in kDa. The S-VP8* P[11] protein was eluted in P3. (C) A micrograph of negative staining transmission electron microscopy (TEM) of the protein from P3 peak shows spheric-shaped PVNPs.

Statistical analyses for Figure 4A:

Coat GST-VP8 P4 protein:
Trivalent vs. S_{60}-VP8 P4: *P*=0.5294
Trivalent vs. S_{60}-VP8 P6: *P*=0.0079
Trivalent vs. S_{60}-VP8 P8: *P*=0.1689

Coat GST-VP8 P6 protein:
Trivalent vs. S_{60}-VP8 P4: *P<0.0001*
Trivalent vs. S_{60}-VP8 P6: *P*=0.5490
Trivalent vs. S_{60}-VP8 P8: *P*<0.0001

Coat GST-VP8 P8 protein:
Trivalent vs. S_{60}-VP8 P4: *P*=0.0055
Trivalent vs. S_{60}-VP8 P6: *P*=0.0004
Trivalent vs. S_{60}-VP8 P8: *P*=0.7560

Coat GST-VP8 P4 protein:
S_{60}-VP8 P4 vs. S_{60}-VP8 P6: *P*<0.0001
S_{60}-VP8 P4 vs. S_{60}-VP8 P8: *P*=0.5349
S_{60}-VP8 P6 vs. S_{60}-VP8 P8: *P*=0.4523

Coat GST-VP8 P6 protein:
S_{60}-VP8 P4 vs. S_{60}-VP8 P6: *P*=0.0003
S_{60}-VP8 P4 vs. S_{60}-VP8 P8: *P*=0.0951
S_{60}-VP8 P6 vs. S_{60}-VP8 P8: *P*<0.0001

Coat GST-VP8 P8 protein:
S_{60}-VP8 P4 vs. S_{60}-VP8 P6: *P*=0.4543
S_{60}-VP8 P4 vs. S_{60}-VP8 P8: *P*=0.0181
S_{60}-VP8 P6 vs. S_{60}-VP8 P8: *P*=0.0025

Statistical analyses for 4B:

Coat GST-VP8 P4 protein:
Trivalent vs. S_{60}-VP8 P4: *P*=0.4835
Trivalent vs. S_{60}-VP8 P6: *P*=0.0006
Trivalent vs. S_{60}-VP8 P8: *P*=0.0238

Coat GST-VP8 P6 protein:
Trivalent vs. S_{60}-VP8 P4: *P*=0.0003
Trivalent vs. S_{60}-VP8 P6: *P*=0.9992
Trivalent vs. S_{60}-VP8 P8: *P*<0.0001

Coat GST-VP8 P8 protein:
Trivalent vs. S_{60}-VP8 P4: *P*=0.0006
Trivalent vs. S_{60}-VP8 P6: *P*=0.0001
Trivalent vs. S_{60}-VP8 P8: *P*=0.9992

Coat GST-VP8 P4 protein:
S_{60}-VP8 P4 vs. S_{60}-VP8 P6: *P*=0.0085
S_{60}-VP8 P4 vs. S_{60}-VP8 P8: *P*=0.1095
S_{60}-VP8 P6 vs. S_{60}-VP8 P8: *P*=0.3687

Coat GST-VP8 P6 protein:
S_{60}-VP8 P4 vs. S_{60}-VP8 P6: *P*=0.0003
S_{60}-VP8 P4 vs. S_{60}-VP8 P8: *P*=0.1390
S_{60}-VP8 P6 vs. S_{60}-VP8 P8: *P*<0.0001

Coat GST-VP8 P8 protein:
S_{60}-VP8 P4 vs. S_{60}-VP8 P6: *P*=0.2656
S_{60}-VP8 P4 vs. S_{60}-VP8 P8: *P*=0.0098
S_{60}-VP8 P6 vs. S_{60}-VP8 P8: *P*=0.0040

Statistical analyses for Figure 5:

Coat GST-VP8 P4 protein:
Trivalent vs. S_{60}-VP8 P4: *P*=0.1965
Trivalent vs. S_{60}-VP8 P6: *P*=0.0085
Trivalent vs. S_{60}-VP8 P8: *P*=0.0444

Coat GST-VP8 P6 protein:
Trivalent vs. S_{60}-VP8 P4: *P*=0.0042
Trivalent vs. S_{60}-VP8 P6: *P*=0.2491
Trivalent vs. S_{60}-VP8 P8: *P*=0.0054

Coat GST-VP8 P8 protein:
Trivalent vs. S_{60}-VP8 P4: *P*=0.0158
Trivalent vs. S_{60}-VP8 P6: *P*=0.0080
Trivalent vs. S_{60}-VP8 P8: *P*=0.2181

Coat GST-VP8 P4 protein:
S_{60}-VP8 P4 vs. S_{60}-VP8 P6: *P*=0.0008
S_{60}-VP8 P4 vs. S_{60}-VP8 P8: *P*=0.0041
S_{60}-VP8 P6 vs. S_{60}-VP8 P8: *P*=0.3668

Coat GST-VP8 P6 protein:
S_{60}-VP8 P4 vs. S_{60}-VP8 P6: *P*=0.0151
S_{60}-VP8 P4 vs. S_{60}-VP8 P8: *P*=0.7384
S_{60}-VP8 P6 vs. S_{60}-VP8 P8: *P*=0.0170

Coat GST-VP8 P8 protein:
S_{60}-VP8 P4 vs. S_{60}-VP8 P6: *P*=0.0391
S_{60}-VP8 P4 vs. S_{60}-VP8 P8: *P*=0.0340
S_{60}-VP8 P6 vs. S_{60}-VP8 P8: *P*=0.0097

Statistical analyses for Figure 6A:

Trivalent vs. S_{60}-VP8 P4: *P*=0.0234

Trivalent vs. S_{60}-VP8 P6: *P*=0.0028

Trivalent vs. S_{60}-VP8 P8: *P*=0.6510

S_{60}-VP8 P4 vs. S_{60}-VP8 P6: *P*=0.1473

S_{60}-VP8 P4 vs. S_{60}-VP8 P8: *P*=0.0304

S_{60}-VP8 P6 vs. S_{60}-VP8 P8: *P*=0.0019

Statistical analyses for Figure 6B:

Trivalent vaccine:
G1P8 Wa vs. G2P4 DS1: *P*=0.7560
G1P8 Wa vs. G4P6 ST3: *P*=0.3195
G2P4 DS1 vs. G4P6 ST3: *P*=0.5413

S_{60}-VP8* P4 vaccine:
G1P8 Wa vs. G2P4 DS1: *P*=0.0246
G1P8 Wa vs. G4P6 ST3: *P*=0.0008
G2P4 DS1 vs. G4P6 ST3: *P*=0.0043

S_{60}-VP8* P8 vaccine:
G1P8 Wa vs. G2P4 DS1: *P*=0.0205
G1P8 Wa vs. G4P6 ST3: *P*=0.0039
G2P4 DS1 vs. G4P6 ST3: *P*=0.0012

S_{60}-VP8* P6 vaccine:
G1P8 Wa vs. G2P4 DS1: *P*=0.4295
G1P8 Wa vs. G4P6 ST3: *P*=0.0009
G2P4 DS1 vs. G4P6 ST3: *P*=0.0009
